# Rib index

**DOI:** 10.1186/s13013-014-0020-9

**Published:** 2014-11-20

**Authors:** Theodoros B Grivas

**Affiliations:** Orthopaedic & Traumatology Department, “Tzaneio” General Hospital of Piraeus, Piraeus, Greece

## Abstract

This article analyzes the double rib contour sign (DRCS) and the rib index (RI). The analyzed topics are 1. the history of presentations – publication of DRCS-RI, 2. the study source origin: school screening for idiopathic scoliosis (IS), 3. what the DRCS and the RI are– Description, 4. the quantification of the DRCS – RI, 5. a reliability study for RI 6. how much the rib index is affected by the distance between the radiation source and the irradiated individual, 7. the implications on IS aetiology, 8. the applications of Rib index for *a. documentation of the deformity, b. assessment of physiotherapy, c. assessment of brace treatment and d. pre- and post-operative assessment*; assessment of the rib-cage deformity correction on the transverse plane, 9. the use of RI and implications for screening policies 10. the reference of the RI method in spinal textbooks and finally 11. the citations in Google Scholar.

## The history of presentation and publication of DRCS-RI

The DRCS and RI were initially presented in Greece during the 25^th^ “Nicolas Giannestras- Panayiotis Smyrnis” Anniversary Symposium of Spinal Column Diseases, at the Porto Rio Hotel, Patras, Greece, 21-23 May 1999 [1]. It was then presented in the International Research Society of Spinal Deformities meeting at Clermont Ferrand, Château du Marand, France, 23-26 May 2000 [2]. Subsequently, it was published in 2002 [3]. This publication focused on the implications of DRCS on the aetiology of idiopathic scoliosis [3].

## The study source origin: school screening for IS

The scoliosis school screening program of the Orthopaedic Department of “Thriasio” General Hospital of Attica started in the school year of 1996. As far as we know, it has been the only program screening children attending the first grade of the primary school aged 5 to 6 years to the last grade of high school aged 17 to 18 years and therefore the collected data covered this wide range of ages. The screened asymmetric children of this program were referred to the scoliosis clinic of that department for further assessment. The children with a scoliometer reading more than 7 degrees were assessed radiographically. The thoracic cage and the spine were assessed for coronal (scoliosis) and sagittal (kyphosis-lordosis) Cobb angle and the segmental left and right RVAs of the rib-cage. In the lateral spinal radiographs of all asymmetric children it was noticed that there was a double rib contour (DRC), an interesting radiologic sign not previously described in the available to us literature. We coined it “double rib contour sign” (DRCS).

## What are the DRCS and the RI – Description

**Figure 1:**
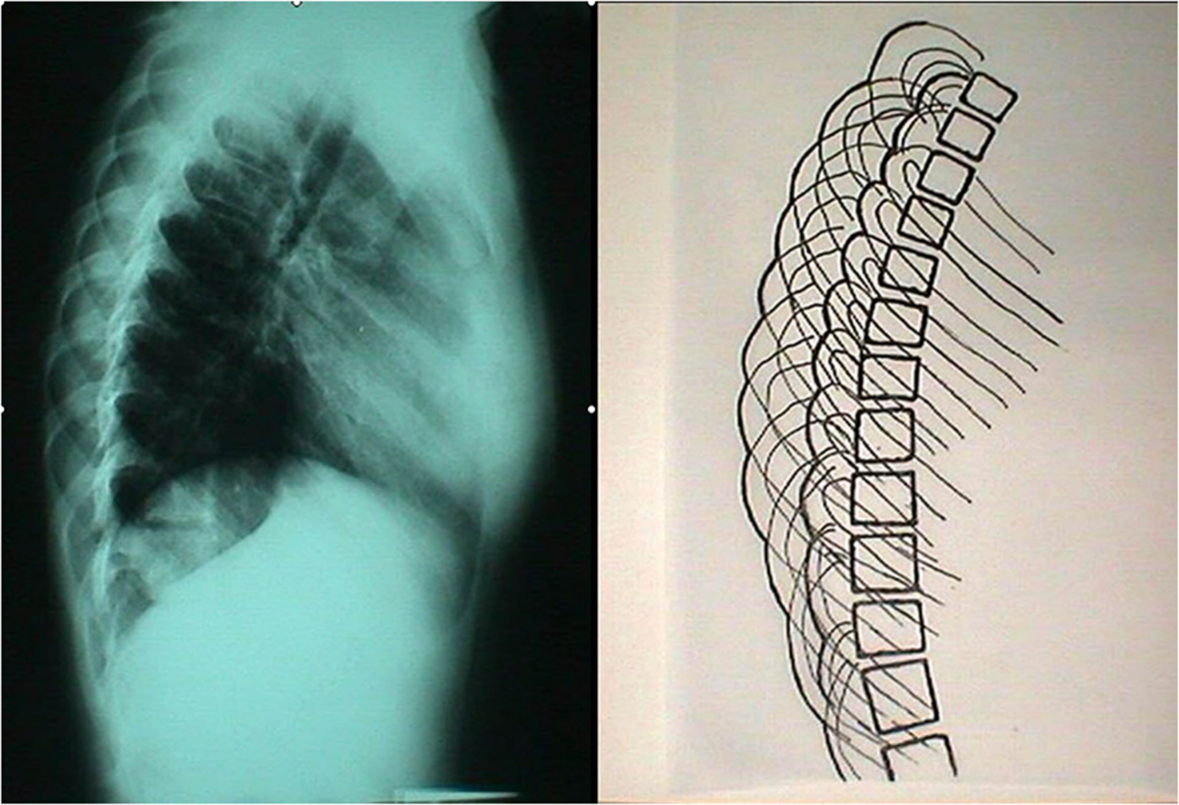
**The double rib contour sign (DRCS) of the thoracic cage in a standing spinal radiograph.**

All lateral spinal radiographs in IS show a DRCS of the thoracic cage, a radiographic expression of the rib hump (RH), Figure 1. The outline of the one hemi-thorax (convex) overlies the contour of the other hemi-thorax (concave). Then the rib index (RI) method extracted from the DRCS was introduced in order to quantify the severity of the double rib contour (DRC) that is to evaluate the rib hump deformity in IS patients in an attempt to create a safe reproducible way to assess the RH deformity based on lateral radiographs. This assessment actually represents the appraisal of the transverse plane rib-cage deformity, a method applied to the lateral spinal radiographs, which has not been presented earlier in the available to us literature, Figure 1.

## Quantification of the DRC sign

For the quantification of the DRCS and formation of the RI we:

1. Determine the most extended rib point; p1. This is the osculation point of a vertical line tangential to the most extended point of the most extending rib contour (convex).
2. Draw a vertical line passing in p1; Line 1.
3. Determine the least projected rib point; p2. This is the osculation point of a vertical line tangential to the most extended point of the least extending rib contour (concave).
4. Draw a vertical line passing in p2; Line 2.
5. Determine the corresponding vertebra and its posterior margin line; Line 3.
6. Measure the distance from line 3 to Line 1 and Line 2.
7. The distance between Line 3 and Line 1 is d1.
8. The distance between Line 3 and Line 2 is d2.

In the lateral radiographs, the posterior body’s margin line of an un-rotated corresponding vertebra represents the Line 3. When the corresponding vertebra is rotated, the posterior margin of the vertebral body shows double line. Then the Line 3 is the parallel one that passes in the middle of this double line.

The corresponding vertebra is the one at the level of d1. We define this in order to draw the Line 3.

Therefore d1 is the distance between the most extended point of the most extending rib contour and the posterior margin of the corresponding vertebra on the lateral scoliosis films, while d2 is the distance from the least projected rib contour and the posterior margin of the same vertebra.

**Figure 2:**
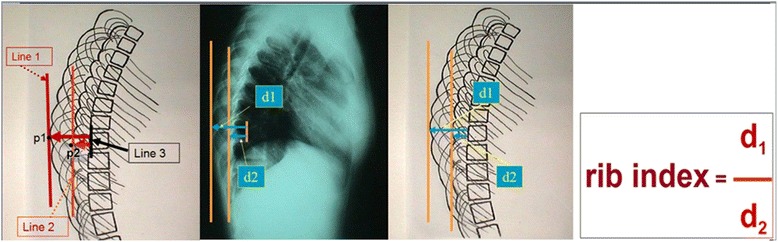
**The Rib Index (RI) of the thoracic cage in a standing spinal radiograph.**

Lastly the “rib index” is defined as the d1/d2 ratio (calculated by the ratio of spine distances d1/d2). At a symmetric and non-deformed thorax the rib contour (RC) lines are practically superimposed and the “rib index” is ≈ 1, Figure 2.

## Reliability study

**Figure 3:**
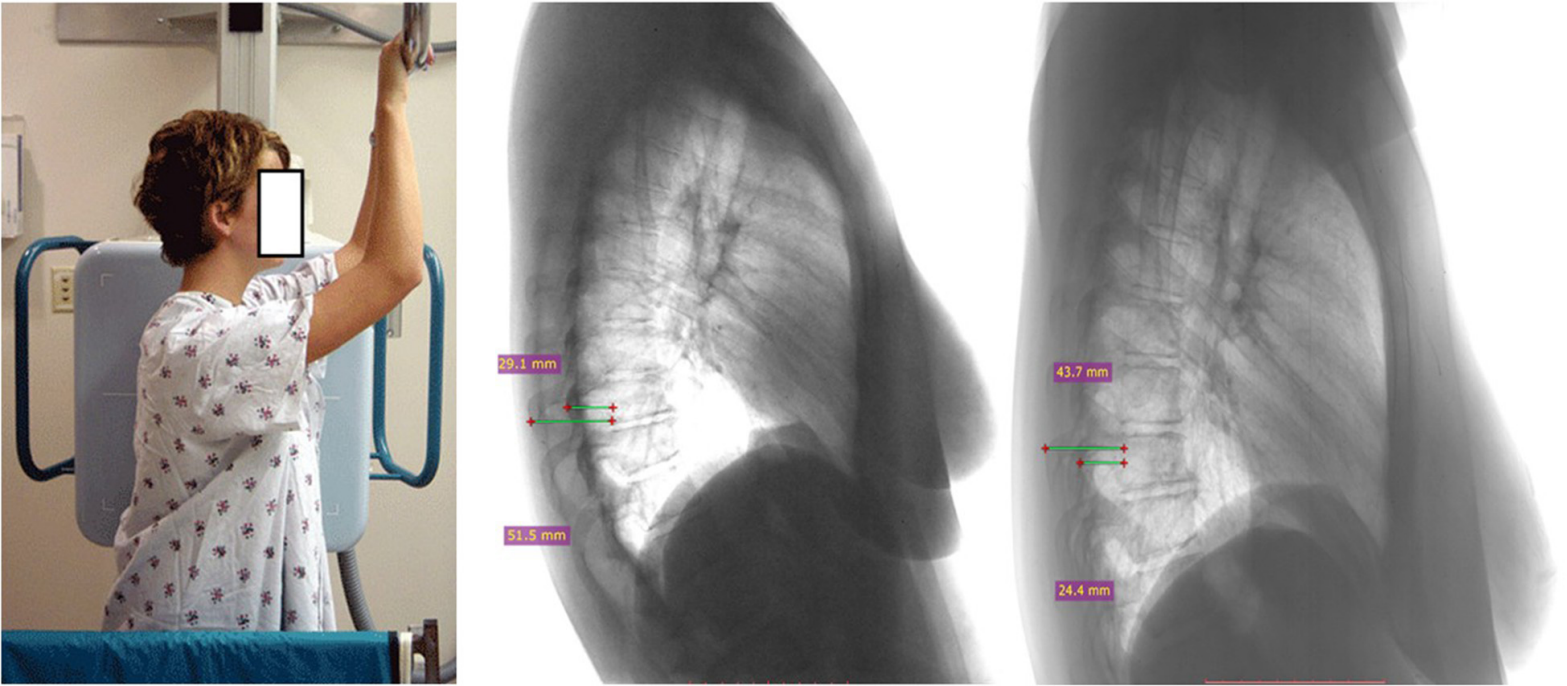
**The radiographs were obtained in a standard position.** Measurements in two radiographs.

The variations of the RI in a pair set of lateral chest radiographs in 49 randomized volunteers were estimated. Each of these subjects had two consequent chest lateral radiographs (LRs) at the radiological department of the hospital by the same technician during the course of treatment (named A and B group of LRs) in a standard position, Figure 3. The cassette was on the left side of the patient. The distance from the radiation source during lateral chest radiographs was chosen to be 1,80m, that is the one used in the radiographs for the scoliotic children according to the American College of Radiology’s recommendations (2009) [4]. The RI was calculated in both LRs of each patient. The statistical analysis included the paired t-test in the set of LRs, its correlation coefficient, the intra- and inter-observer error using the formula (SD/√2)/2, where SD is this of the differences of the two sets of measurement (As-Bs). The SPSS v16 statistical package was used. It was found that in the 49 pairs of LRs there was no statistical difference of the RI, (paired t-test p < 0.314). The RI in the A and B group of LRs was highly correlated (correlation coefficient R = 0,924, p < 0.0001). The intra-observer error was 0.0080, while the inter-observer error was 0.0213, in terms of 95% confidence interval (CI). Therefore the RI proves to be a reliable method to evaluate the thoracic deformity or the effect of surgical or non-operative treatment on the IS rib-cage deformity (hump). It was also demonstrated that the RI is a simple method and a safe reproducible way to assess the RH deformity based on lateral radiographs, without the need for any other special radiographs and exposure to additional radiation [5].

Ethical approval was provided for this study by the Ethical Committee of the "Attikon" General University Hospital of Athens, Greece". This study was in compliance with the Helsinki Declaration.

## How much is the RI affected by the distance between the radiation source and the irradiated individual?

A validity study of DRCS was implemented to appraise how the RI is affected by the distance between the radiation source and the irradiated child.

**Figure 4:**
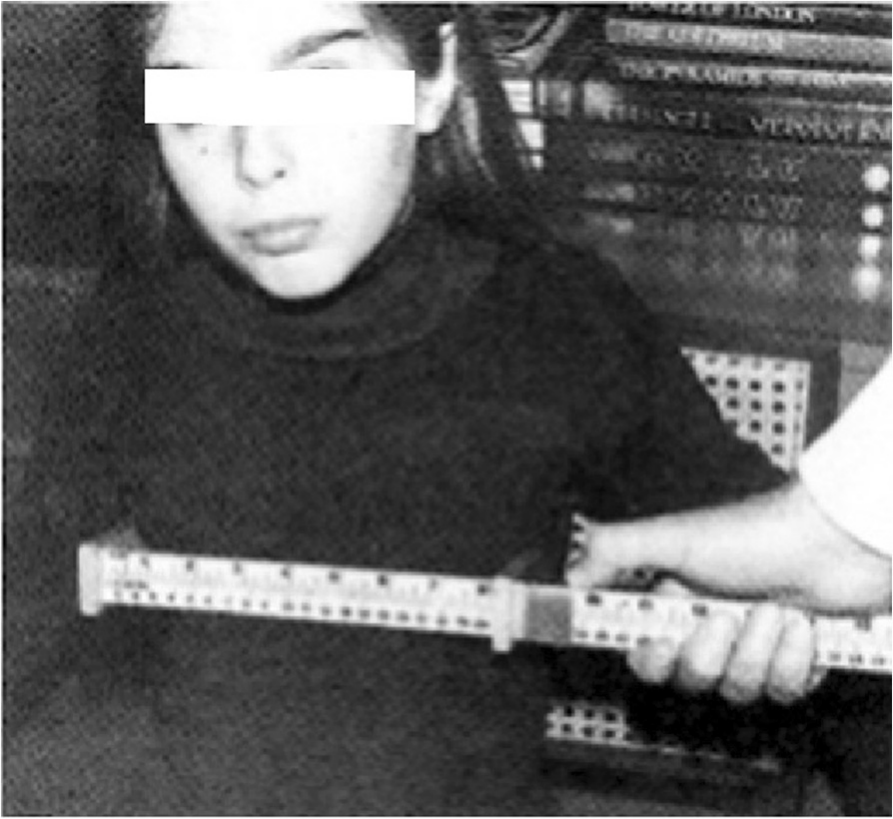
**The way the transverse diameter of the rib-cage is documented in a child using a special anthropometric tool.**

**Figure 5:**
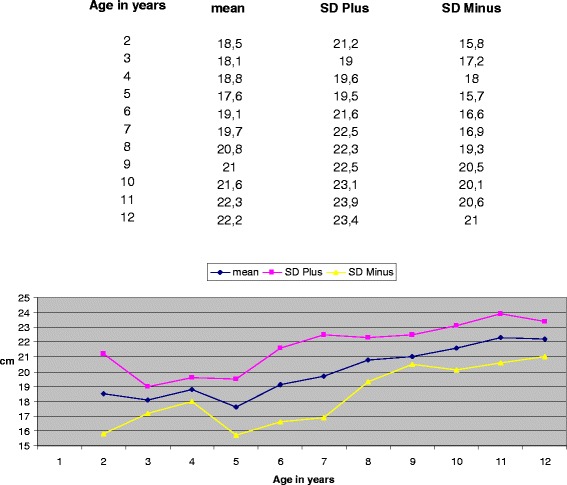
**Transverse thoracic diameter values in cm for boys by age.**

**Figure 6:**
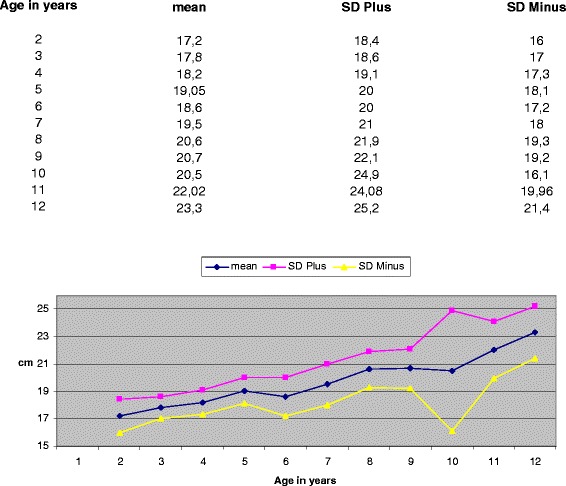
**Transverse thoracic diameter values in cm for girls by age.**

**Figure 7:**
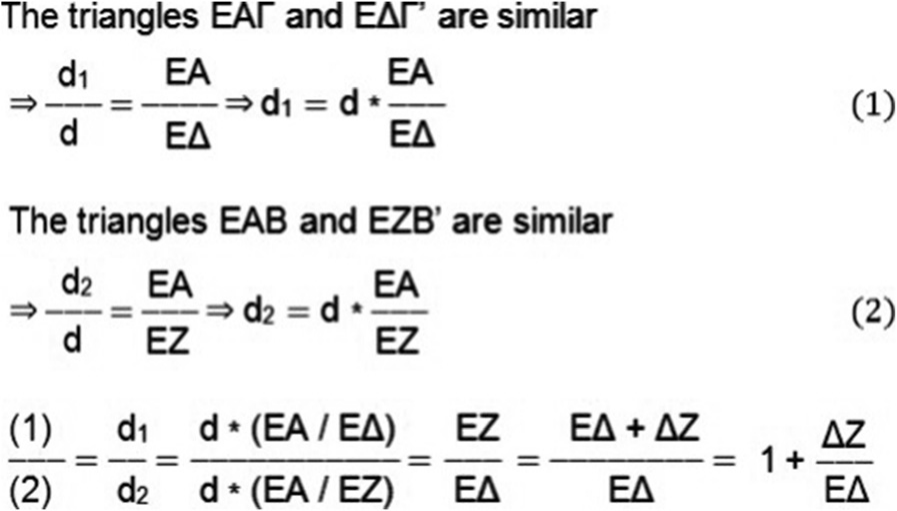
**The Euclidean geometry used for the assessment of d1/d2 quotient.**

**Figure 8:**
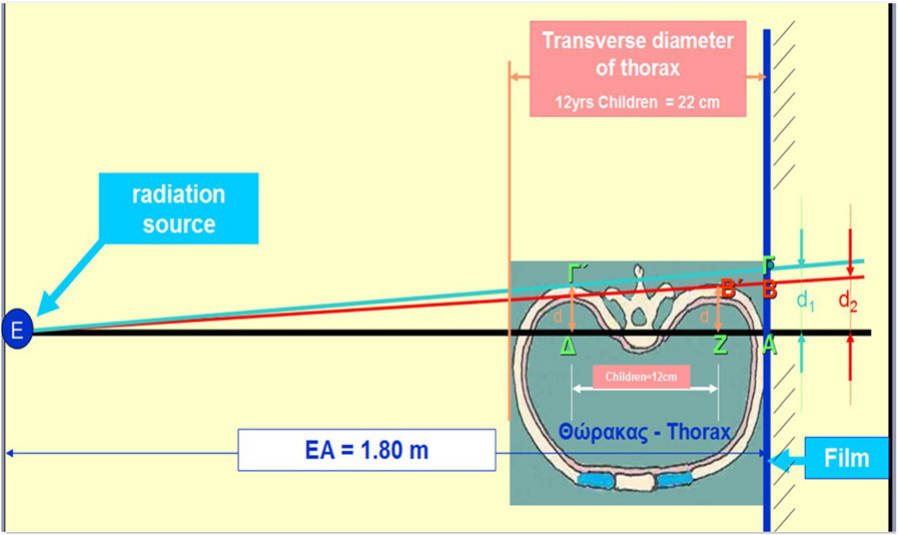
**The set up for obtaining the radiograph and the used points (Greek letters) for the assessment of d1/d2 quotient.**

The American College of Radiology's (2009) guidelines for obtaining radiographs for scoliosis in children recommend that the distance from the scoliotic child to the film is 1,80 m [4]. Normal values for the transverse diameter of the ribcage in children aged 6-12 years were considered those reported by Grivas in 1988 (Figures 4, 5 and 6) [6]. According to the Euclidean geometry (Figure 7), d1/d2 = 1.073 at a normal child 12 years of age, provided that the distance ΔZ ≈ 12cm (11,84) and EA = 180cm, with transverse rib-cage diameter of the child 22cm (Figure 8). The values of the transverse rib-cage diameter measurements are presented in centimeters by age (2-12 years old) in boys (Figure 5) and in girls (Figure 6).

This validity study demonstrated that the DRCS is substantially true and practically the RI is not affected by the distance between the radiation source and the irradiated child [7].

However the positioning of the IS patient for the radiographic examination is very important. Each radiograph must be taken by technicians trained to do radiography in a standard way in any hospital or laboratory.

The 1,8m distance between the radiation source and the irradiated individual may vary, depending on the examinee's body size. In order to eliminate this limitation, we calculated the d1/d2 ratio using a couple of distances (1,9m and 1,7m) between the radiation source and the irradiated child. This variation corresponds to different body sizes (1,9m for larger/fatter and 1,7m for smaller/thinner children respectively). The results were almost similar and statistically insignificant. In conclusion, body size was found irrelevant in the measurement of the RI.

## Implications on aetiology of IS

At the introduction it was noted that the screened asymmetric children of our screening program, with equal or more than 7 degrees of scoliometer reading, were referred to the scoliosis clinic for further assessment. In a cohort of 133 similar consequent children spinal postero-anterior and lateral radiographs were obtained, [1-3]. The DRCS was assessed in children with and without late onset idiopathic scoliosis (LOIS) with 10° - 20° Cobb angle and it was examined whether the deformity of the thorax or that of the spine develops first in IS. The cohort of children under investigation included 47 boys with a mean age 13.28 years and 86 girls with mean 13.39 years. All of them had a scoliometer reading (Angle of trunk rotation or inclination ATR or ATI) ATI ≥7°.

The Cobb angle was also assessed. Five groups of children were formed, as described below:

- **group 1** children with no scoliosis and *straight spines*
- n = 27 (12 boys 15 girls)
- **group 2** children with no scoliosis, *spinal curvature having*
- *a Cobb angle 1° - 9°*
- n = 13, (6 boys +7 girls)
- **group 3** children with *thoracic scoliosis 10° - 20°*
- n = 47 (17 boys +30 girls)
- **group 4** children with *thoracolumbar scoliosis* 10° - 20°
- n = 14 (4 boys +10 girls)
- **group 5** children with *lumbar scoliosis 10*° - 20°
- n = 28 (7 boys +21 girls)

The statistical analysis was done using the SPSS-PC v10 package using the following techniques: frequencies, descriptive (mean, range, min, max, sd dev, sd err, kurtosis, skewness), ANOVA, Kruskal-Wallis test, scatter plot, Pearson Correlation coefficient and independent Samples T-test. The mean RI in **group** 1 was 1.45, in **group 2** = 1.51, in **group 3** = 1.56, in **group 4** = 1.59 and in **group 5** = 1.47. Interestingly, no RI sex differences for boys and girls were found and there was no correlation of the Cobb angle with the RI for the thoracic, thoraco-lumbar and lumbar mild LOIS groups (scoliosis 10°-20° Cobb angle). It was recognized that in all school-screening referrals (having ATI ≥7°), the thoracic deformity in terms of the DRCS has already been developed. It was also interesting to note that there were 20% of children with straight spines, 10% of children having curves with Cobb angle 1°-9°, and 70% of children suffering IS. The non-scoliotics were followed due to the existing RH; they were 1,5-2 years younger than the ones who had already developed IS and they had both a RI ≈ 1,5. The DRCS was present in all referrals; in contrary there was no scoliotic spine without it, as the DRC sign is always present in scoliotic lateral spinal radiographs with no exception. This study led us to the conclusion that the DRCS is primarily the result of rib deformation and secondarily of vertebral rotation, because DRCS could be present in straight spines with no vertebral rotation. This observation supports the hypothesis that in IS the deformity of the thorax develops first and the deformity of the spine succeeds [1-3,8].

Longitudinal follow-up results of data from school screening strengthen this statement [9].

## Applications of Rib index

The RI can be applied for four purposes in the treatment of scoliosis. They are:

- *Documentation of the deformity*
- *Assessment of Physiotherapy*
- *Assessment of brace treatment*
- *Pre-and Postoperative assessment*

### Documentation of the deformity

Using the RI we can assess and document the transverse plane thoracic deformity from lateral spinal radiographs.

### Assessment of Physiotherapy

The RI, as an objective measure to document changes of the RH deformity, was used in non-operative treatment of IS using specific scoliosis physiotherapeutic exercises, (SSPE). Lebel and Lebel reported on a Risser 4 progressive AIS patient treated with the Schroth Method [10].

The authors reported: At diagnosis, the mean RI was 1.658 and the Cobb angle was 45°. In August 2012, the mean RI and Cobb angle increased to 2.352 and 56° respectively, indicating an increase in RH deformity and progression of scoliosis. In November 2012, after 12 weeks of SSPE treatments, the mean RI and Cobb angle started to decrease to 2.16 and 52°. In October 2013, after a total of twelve 2-3hr clinic visits and 12 months of a daily 1.5-2hr Schroth method SSPE, the mean RI decreased to 1.665 and the Cobb angle to 42°. These mean RI and Cobb angle measurements show progression from 2011-2012 and improvement from 2012-2013 as a result of daily SSPE. The authors concluded that the RI can be used as an additional objective measure to show RH improvement with SSPE [10].

### Assessment of brace treatment

The RI was used to assess the initial correction of the RH in AIS treated with the Dynamic Derotation Brace (DDB). It is well established that scoliotic children and their parents are very much concerned about their trunk deformity (TD). One of their TD components is the RH, which is mainly the expression of the rib deformity. The brace treatment aims not only to hold or correct the central axis, the spinal deformity, but also the TD in the thorax, the RH.

**Figure 9:**
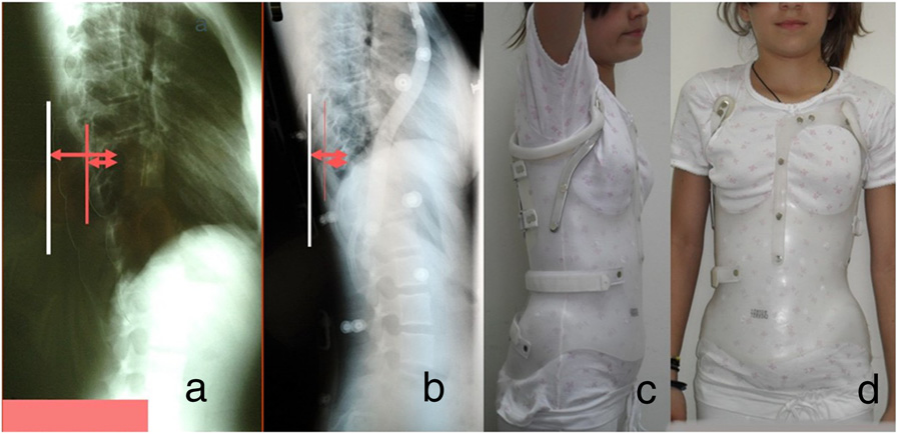
**Assessment of the pre-brace treatment RI and the early post-brace.** Figure **a,b** depicts the radiographs and Figure 9 **c,d** the clinical picture of the girl wearing the brace.

Twenty children with right thoracic (n = 14) and double curves (n = 6) (right thoracic left lumbar) were assessed. The SRS/SOSORT inclusion criteria for brace treatment were used, [11-14]. The reference vertebra from which the RI was assessed was documented. Statistical analysis was done using the t-test. The mean thoracic Cobb angle was 27, 5 degrees. The posterior margin of the reference vertebra was the T8 in 4 scoliotics, T9 in 2, T10 in 4, T11 in 6, L1 in 2 and L2 in 2, respectively. The mean pre-brace treatment RI was 1,864 and the early post-brace 1,205 respectively, p = 0,007. It was concluded that the DDB significantly improves the RH deformity during the initial treatment period in the thoracic curves and in the thoracic component of the double scoliotic curves (Figure 9), [15,16].

### Pre-and Postoperative assessment

The assessment of the post-operative rib-cage deformity correction on the transverse plane, using various surgical techniques, was recently reported [17-20].

The first publication of a follow-up study on surgically treated patient, in which the RI was applied to assess rib hump correction, was by A. Crawford and his group in 2012 [17]. It was initially used in a multi-center study for AIS of Lenke type I curves treated with posterior spinal fusion with or without costoplasty, instrumented with pedicle screws or hybrid constructs, with a minimum follow-up of 2 years. The first group (Group I) was treated with pedicle screws, direct vertebral rotation and no costoplasty, while the second group (Group II) was treated with pedicle screws, vertebral rotation and costoplasty. The RI calculated from the DRCS was measured radiographically and compared between the two groups. The statistical analysis of pre-operative and post-operative RH deformity correction using the RI showed that costoplasty combined with pedicle screws and vertebral derotation may significantly improve rib hump deformity, as opposed to pedicle screws and vertebral derotation alone, [17].

Subsequently Stavropoulos et al 2014 measured the RH deformity correction in AIS treated with full screw or hybrid constructs using the DRCS and RI. Sixteen scoliotics (group A) were operated upon using full pedicle screw with median age 15 years and 9 scoliotics (group B) were operated using hybrid construct with median age 17.2 years. RI correction was calculated by subtracting the postoperative RI from the preoperative RI. In group A the mean pre-operative RI was 1.93 and the post-operative 1.37 (p < 0.001). In group B the mean pre-operative RI was 2.06, while the mean post-operative RI was 1.5 (p = 0.008). However, between group A and B the RI correction means was not found to be statistically significant, p = 0.803. The authors concluded that the RH deformity correction is the same, no mater what type of spinal construct is used. They also stated that, according to their results, it is implied that the RH deformity more likely results from asymmetric rib growth rather than from vertebral rotation [18,19].

Haber et al also used the RI, in an effort to provide some more insight on the continuing debate whether all-screw constructs outperform hybrid constructs in small, flexible thoracic AIS. They endorse the statement that “The RI has also been shown to be a reliable method to judge rib rotation owing to its simplicity and ability to be measured on lateral film” [20].

## The use of RI and implications for screening policies

The introduction of the RI resulted in a very important knowledge derived from the statistical analysis of the correlation of Cobb angle to the RI in radiographs from the screening programs referrals.

**Figure 10:**
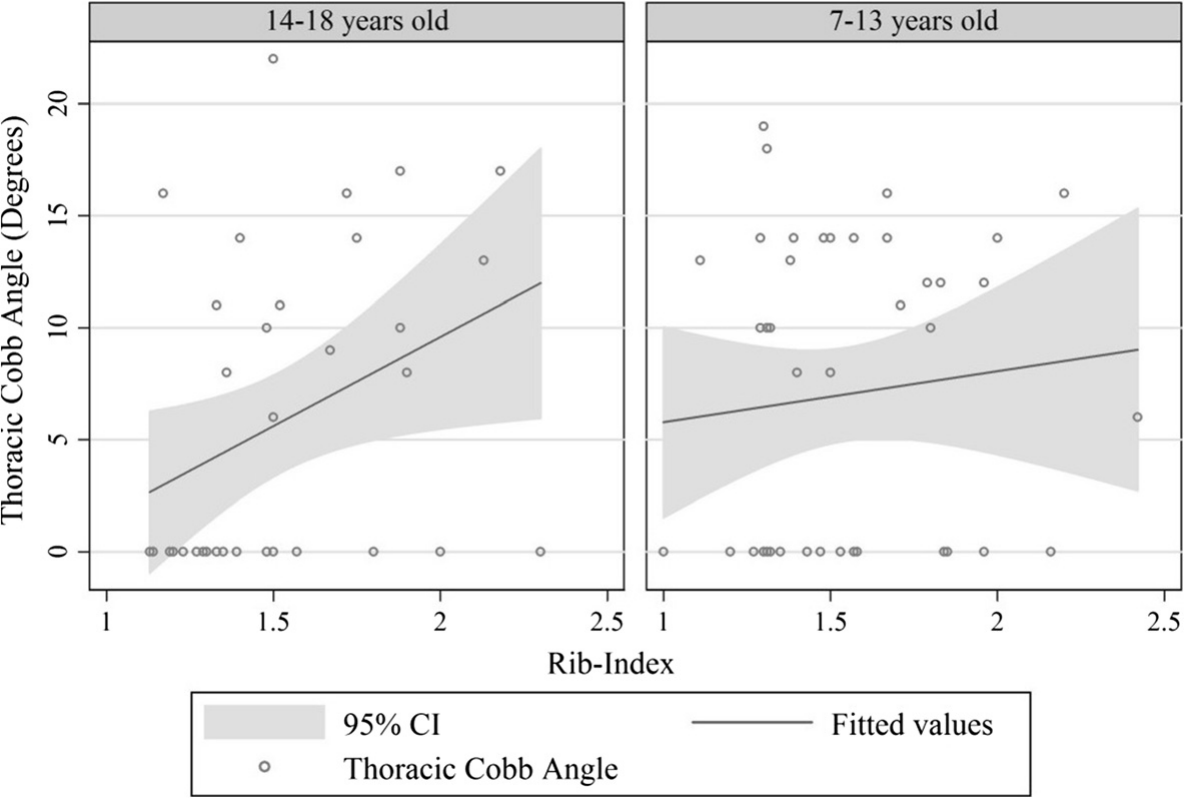
**The linear relationship between thoracic Cobb angle and RI is graphically depicted.** There is only linear association between thoracic Cobb Angle and rib-index in the age group of 14–18 years. (Predicted Thoracic Cobb Angle = - 6.357 + 7.974 Χ (Rib-Index) [16].

Approximately 30% of younger referred girls, aged equal or less than 13 years old with an ATR ≥7°, were found to have either a straight spine or a spinal curve under 10°. In this age group the correlation between thoracic deformity in terms of RH (the transverse plane thoracic deformity) assessed using the RI and the radiographic spinal measurement in terms of Cobb angle, is not statistically significant, while in older referred girls, aged 14–18 years old, it is [21]. Thus, all younger individuals, referred to scoliosis clinics from screening programs, who are identified with a surface deformity (RH) but without a severe scoliotic curve (Cobb angle), are at risk to develop IS. All these children should be followed and not discharged from regular follow-up, see Figure 10. In line with this statement are the Nissinen et al 1993 school screening longitudinal study findings [9].

Without the aforementioned screening program that included younger children in addition to the usual age ranges, the above statement would not be made [22]. The aim of school screening is to identify most or all the individuals with unrecognized IS at an early stage, when a less invasive treatment is more effective. It was earlier emphasized that a further contribution of the screening programs is in the research of IS epidemiology, natural history and aetiology [22]. In addition, these programs are a unique tool for research of IS in humans. In most published articles all aetiopathogenetic factors are studied in animals and not in humans, in contrast with screening programs where any knowledge on IS aetiology is derived from humans, in other words it is human evidenced based knowledge [23]. Such contribution is beyond the original aim of school screening; however it is very important because we can expand our knowledge and adequately understand the pathogenesis of IS. The role of biological factors such as the menarche, the lateralization of the brain, the handedness, the thoracic cage, the intervertebral disc, the melatonin secretion, as well as the role of environmental factors such as the light and the impact of the geographical latitude in IS prevalence, were studied in children referred from school screening [22]. The current evidence supports that school screening programs should be continued not only for early detection of IS but also as a basis for epidemiological surveys until we learn much more about the aetiology of IS.

The role and usefulness of RI in the school screening program, at a first consideration, seems to be minor in the screening program itself, but very important for research purposes. However, the introduction of RI and its correlation to the Cobb angle of the radiographs of the children referred from school screening, shaped the age range of them who must be followed, so that our decision on this issue was more thorough. Therefore the importance of RI in the school screening program is significant.

## Reference of the RI method in spinal textbooks

A safe way to recognize a method’s importance is to notice if it is referred in pertinent classical text books. The RI method used to assess the RH, in other words the transverse plane thoracic deformity, is currently referred in some classical spinal text books [24,25].

## Citations in Google Scholar

For the article – the first publication on DRCS and RI - The Double Rib Contour Sign (DRCS) in Lateral Spinal Radiographs. Aetiologic Google Scholar provides 45 references (search on 29/8/2014), see below:

- The Double Rib Contour Sign (DRCS) in Lateral Spinal Radiographs. Aetiologic
- **TB GRIVAS**, S Dangas, BD Polyzois… - Research Into Spinal …, 2002 - books.google.com
- Abstract: All lateral spinal radiographs in idiopathic scoliosis show a DRC sign of the thoracic cage, a radiographic expression of the rib hump. The outline of the convex overlies the contour of the concave ribs. The aim of this study is to assess this DRC sign in children**.**
- Γίνεται αναφορά σε 45 Σχετικά άρθρα Όλες οι 6 εκδοχές Παράθεση

## Summary

In this article the DRCS and the RI are analyzed. The history of presentations and the first publication of DRCS-RI are reported. The original source of the study was the referred children for IS from our school screening program; the description and quantification of the DRCS and the RI are explained. The reliability study for RI and how much the RI is affected by the distance between the radiation source and the irradiated individual is another topic of this article. The implications of the DRCS and RI in the aetiology of IS are analyzed. The clinical applications of RI in the assessment of the thoracic deformity in the transverse plane, the outcomes of physiotherapy, bracing and surgical treatment are described. The use of RI and implications for screening policies are also detailed. Finally, the spinal text books referring this method and the Google Scholar citations are enumerated.

## Consent

Written informed consent was obtained from the patient’s guardian/parent/next of kin for the publication of this report and any accompanying images.
